# Efficacy of oleylphosphocholine in experimental cutaneous leishmaniasis

**DOI:** 10.1093/jac/dkad162

**Published:** 2023-05-25

**Authors:** Katrien Van Bocxlaer, Jodie Dixon, Johannes J Platteeuw, Dennie Van Den Heuvel, Kerri-Nicola Mcarthur, Andy Harris, Mo Alavijeh, Simon L Croft, Vanessa Yardley

**Affiliations:** Department of Biology, York Biomedical Research Institute, University of York, York, UK; Department of Biology, York Biomedical Research Institute, University of York, York, UK; Avivia BV, Novio Tech Campus, Nijmegen, The Netherlands; Avivia BV, Novio Tech Campus, Nijmegen, The Netherlands; Pharmidex Pharmaceutical Services Ltd., London, UK; Pharmidex Pharmaceutical Services Ltd., London, UK; Pharmidex Pharmaceutical Services Ltd., London, UK; London School of Hygiene & Tropical Medicine, Faculty of Infectious and Tropical Diseases, London, UK; London School of Hygiene & Tropical Medicine, Faculty of Infectious and Tropical Diseases, London, UK

## Abstract

**Objectives:**

Cutaneous leishmaniasis (CL) is a neglected tropical disease causing a range of skin lesions for which safe and efficacious drugs are lacking. Oleylphosphocholine (OLPC) is structurally similar to miltefosine and has previously demonstrated potent activity against visceral leishmaniasis. We here present the *in vitro* and *in vivo* efficacy of OLPC against CL-causing *Leishmania* species.

**Methods:**

The antileishmanial activities of OLPC were evaluated and compared with miltefosine *in vitro* against intracellular amastigotes of seven CL-causing species. Following the confirmation of significant *in vitro* activity, the performance of the maximum tolerated dose of OLPC was evaluated in an experimental murine model of CL followed by a dose–response titration and the efficacy evaluation of four OLPC formulations (two with a fast-release and two with a slow-release profile) using bioluminescent *Leishmania major* parasites.

**Results:**

OLPC demonstrated potent *in vitro* activity of the same order as miltefosine in the intracellular macrophage model against a range of CL-causing species. A dose of 35 mg of OLPC/kg/day administered orally for 10 days was well-tolerated and able to reduce the parasite load in the skin of *L. major*-infected mice to a similar extent as the positive control paromomycin (50 mg/kg/day, intraperitoneally) in both *in vivo* studies. Reducing the dose of OLPC resulted in inactivity and modifying the release profile using mesoporous silica nanoparticles led to a decrease in activity when solvent-based loading was used in contrast to extrusion-based loading, which had no impact on its antileishmanial efficacy.

**Conclusions:**

Together, these data suggest that OLPC could be a promising alternative to miltefosine treatment for CL. Further investigations exploring experimental models with additional *Leishmania* species and skin pharmacokinetic and dynamic analyses are required.

## Introduction

Cutaneous leishmaniasis (CL) is a neglected tropical disease caused by the obligate intracellular protozoan *Leishmania* parasite. The infection is primarily located in the dermal layers of the skin and manifests as a variety of skin lesions ranging from closed nodules to plaques, ulcers and gross mucosal destruction. CL affects approximately 12 million people,^1^ particularly in certain eco-epidemiological foci that reflect human activities such as migration, urbanization and deforestation characterized by underlying factors of poverty, conflict and climate change. While it does not cause fatalities, the disfiguring lesions and scars lead to stigmatization, reduced quality of life, discrimination and mental health issues, particularly in children and young women.^2,3^

The recommended drugs for the treatment of CL are substandard. The pentavalent antimonials [sodium stibogluconate (Pentostam, SSG) and meglumine antimoniate (Glucantime)] have been the mainstay CL treatment since the 1940s even though they demonstrate variable efficacy across the different CL-causing species and require multiple treatment courses prior to obtaining cure.^4,5^ Miltefosine is an orally active antileishmanial drug and, similar to the antimonials, it displays variable efficacy across the different *Leishmania* species.^6^ In addition, miltefosine is teratogenic and known to cause nausea due to irritation of the gastrointestinal epithelium, which is especially troublesome given the extensive treatment duration (28 days for CL).^7^ Besides these limitations, patient access to miltefosine is severely restricted due to supply chain-induced shortages and lack of affordability with a cost that exceeds far beyond what patients can afford ($57 600 or 3000–12 000 euros for a 28 day treatment in the USA and Europe, respectively)^8^ despite an initial agreement between the manufacturer and public institutions.^9,10^

Oleylphosphocholine (OLPC), an alkylphosphocholine with structural similarities to miltefosine, was developed in an attempt to overcome some of the treatment limitations mentioned above. The activity of the drug was first evaluated against visceral leishmaniasis (VL) and whilst it demonstrated similar *in vitro* EC_50_ values compared with miltefosine in the intracellular amastigote model, OLPC performed markedly better than miltefosine in the early curative VL model in hamsters.^11^ Five doses of 20 mg/kg/day OLPC resulted in 91% and 99% parasite load reduction in the liver and spleen compared with 0% and 61% for miltefosine, respectively. Higher doses of OLPC (5 doses of 40 mg/kg/day) were well tolerated and resulted in even more pronounced parasite load reductions in the target organs (liver, spleen and bone marrow).^11,12^ Given a similar parasite susceptibility to OLPC and miltefosine, the enhanced *in vivo* efficacy against VL was hypothesized to be related to pharmacokinetics, a hypothesis that upon more detailed investigation revealed a 35% increased bioavailability and an earlier *T*_max_ for the liposomal formulation of OLPC suggesting enhanced absorption either through passive or active processes.^12^ In dogs, a proof-of-concept study revealed that a short course (14 days) of 4 mg/kg OLPC reduced parasite numbers in bone marrow aspirates and improved clinical manifestations of canine leishmaniosis.^13^ Due to the lack of a miltefosine treatment group, the direct comparison of both treatments was not possible. In an experimental model of CL using transgenic *Leishmania major*, cutaneous parasite burdens reduced 34% and 93.5% upon treatment with OLPC (40 mg/kg/day) compared with the vehicle control group after 5 and 10 days of drug administration, respectively.^14^ A detailed investigation of the skin parasite load and drug concentration is still lacking.^13^

Whereas these *in vivo* studies demonstrate a superior performance of OLPC against *Leishmania* parasites situated in both visceral organs and skin compared with miltefosine, certain physicochemical properties, in particular the low melting point (T_m_  ~55°C) and hygroscopic nature^15^ of OLPC, complicate the pharmaceutical development process. In addition, the double bond present in the alkyl chain of OLPC is prone to oxidation. Excipients are typically introduced to streamline the manufacturing process and guarantee the stability and optimal bioavailability of the active pharmaceutical ingredient (API) in the final pharmaceutical form (e.g. tablet, capsule). Therefore, we report here, for the first time to our knowledge, a strategic approach allowing the head-to-head comparison of the newly developed pharmaceutical formulations with the API in an experimental model of CL early on in the development process. The antileishmanial potency of the API (OLPC as such) against seven CL-causing species in the intracellular amastigote model was established followed by a dose–response titration of OLPC versus miltefosine in an experimental *L. major* model. The most effective dosing regimen was then selected to compare the ability of several OLPC formulations using diverse excipients and release profiles (two with a fast-release and two with a slow-release profile) to reduce the skin parasite burden.

## Materials and methods

### Drugs and formulations

OLPC and miltefosine (API as such) were kindly provided by Oblita Therapeutics (Zoersel, Belgium) and Paladin Lab Inc. (Montréal, Canada), respectively. For the *in vitro* antileishmanial activity evaluation, stock solutions were prepared to a concentration of 20 mM in phosphate buffered saline (PBS; 0.9% NaOH, pH 7.4; Sigma–Aldrich, UK), filter sterilized and stored at −20°C until use. Amphotericin B deoxycholate (Fungizone, E.R. Squibb & Sons, UK) was purchased from the York Hospital pharmacy (York, UK) and prepared to a concentration of 5.4 µM as per the manufacturer’s instructions.

OLPC formulations were developed using standard excipients including lactose (OLH.200511) and microcrystalline cellulose (MCC; OLH.200616) and resulted in an instant and almost complete drug release in deionized water (>90% OLPC release; Table 1). To modify the physicochemical properties of OLPC for formulation purposes and potentially reduce the irritation of the gastrointestinal epithelium, OLPC was encapsulated in mesoporous silica particles using either solvent-based loading (ethanol; OLH.200403) or extrusion-type milling (solvent-free loading; OLH.200415).^16^ Both formulations demonstrated a delayed and incomplete release of the drugs (approximately 55%–58% OLPC release; Table 1). For the *in vivo* experiments, the miltefosine and OLPC-containing formulations were prepared daily by dissolving the powder in sterile PBS to the respective concentrations indicated in Table 2. Formulations were administered within 5 min of preparation. Paromomycin sulphate (Sigma–Aldrich, UK) was equally prepared in sterile PBS to yield 50 mg of paromomycin/kg and stored at 4°C throughout the drug treatment. d- Luciferin was solubilized in Dulbecco’s PBS (without calcium and magnesium) at a concentration of 30 mg/mL, subsequently filtered (0.2 μM) and stored at −20°C until required.

**Table 1.** The different OLPC formulations and the release profile in deionized water

Batch number	Excipient	% OLPC release (deionized water—1 h)	Release profile
OLH.200616.01-01	MCC ph102	95	Fast release
OLH.200511.03-01	Lactose	92	Fast release
OLH.200403.01-01	Silica 3150 (EtOH)	55	Slow release
OLH.200415.01-01	Silica 3150 (Extr)	58	Slow release

MCC, microcrystalline cellulose; EtOH, ethanol; Extr, extrusion.

**Table 2.** Drug formulation details and dose regimens for *in vivo* study 1 and 2

Group ID	Mice per group	Drug	Dose—concentration	Vehicle	Administration Route	Frequency of dosing
*In vivo* study 1^a^						
* *Group 1	5	Untreated control	N/A	N/A	N/A	N/A
* *Group 2	5	Paromomycin	50 mg/kg–5 mg/mL	Sterile PBS	IP	1/day
* *Group 3	8	Miltefosine	35 mg/kg–3.5 mg/mL	Sterile PBS	Oral	1/day
* *Group 4	8	OLPC	35 mg/kg–3.5 mg/mL	Sterile PBS	Oral	1/day
*In vivo* study 2^a^						
* *Group 1	5	Untreated control	N/A	N/A	N/A	N/A
* *Group 2	5	Paromomycin	50 mg/kg–5 mg/mL	Sterile PBS	IP	1/day
* *Group 3	5	Miltefosine	35 mg/kg–3.5 mg/mL	Sterile PBS	Oral	1/day
* *Group 4	5	Miltefosine	17.5 mg/kg–1.75 mg/mL	Sterile PBS	Oral	1/day
* *Group 5	5	OLPC	35 mg/kg–3.5 mg/mL	Sterile PBS	Oral	1/day
* *Group 6	5	OLPC	17.5 mg/kg–1.75 mg/mL	Sterile PBS	Oral	1/day
* *Group 7	5	OLPC	8.75 mg/kg–0.875 mg/mL	Sterile PBS	Oral	1/day
* *Group 8	5	OLPC–OLH.200403^b^	Eq 35 mg/kg–eq 3.5 mg/mL	Sterile PBS	Oral	1/day
* *Group 9	5	OLPC–OLH.200415^b^	Eq 35 mg/kg–eq 3.5 mg/mL	Sterile PBS	Oral	1/day
* *Group 10	5	OLPC–OLH.200511^b^	Eq 35 mg/kg–eq 3.5 mg/mL	Sterile PBS	Oral	1/day
* *Group 11	5	OLPC–OLH.200616^b^	Eq 35 mg/kg–eq 3.5 mg/mL	Sterile PBS	Oral	1/day
* *Group 12	5	Blank–naive mice	N/A	N/A	N/A	N/A

N/A, not available.

Treatment was administered for 10 consecutive days.

A new formulation was prepared each day by adding sterile PBS to the powder just prior to drug administration.

### Parasites

The two strains of *L. major* parasites [(MHOM/SA/85/JISH118 and Ppy RE9H + *L. major* Friedlin (MHOM/IL/81/Friedlin)] and one of *Leishmania mexicana* (MNYC/BZ/62/M379) were extracted from BALB/c mouse skin and left to transform into promastigotes in Schneider’s medium with 10% FCS at 26°C. The remaining strains [*Leishmania tropica* (MHOM/IR/2013/HTD4), *Leishmania aethiopica* (MHOM/ET/84/KH), *Leishmania panamensis* (MHOM/PA/71/LS94), *Leishmania braziliensis* (MHOM/BR/75/M2903) and *Leishmania amazonensis* (clinical isolate HTD13)] were left in M199 medium (Gibco) with 10% heat-inactivated FCS and used within eight *in vitro* passages.

### Ethics

All animal work was carried out under a UK Home Office project licence according to the Animal (Scientific Procedures) Act 1986 and the European Directive 2010/63/EU. The project licence (PPL P00B3B595) was reviewed by the University of York Animal Welfare and Ethical Review Board prior to submission and consequent approval by the UK Home Office.

### Intracellular amastigote drug susceptibility evaluation

Peritoneal macrophages (PEMs) were collected by lavage from the abdominal cavity of female CD1 mice (7–10 weeks old) with RPMI-1640 24 h after induction with 2% starch in sterile PBS. The PEMs were then washed, counted and resuspended in RPMI-1640 medium supplemented with 10% FCS. Aliquots containing 4 × 10^4^ PEMs were transferred to 16-well LabTek slides and left overnight at 37°C in an atmosphere of 5% CO_2_ in air. The next day, *Leishmania* parasites resuspended in RPMI-1640 with 10% FCS medium were added to the macrophages in a 3:1 (*L. major*, *L. amazonensis*), 5:1 (*L. mexicana*, *L. tropica*) or 7:1 (*L. aethiopica*, *L. braziliensis*, *L. panamensis*) ratio.

Twenty-four hours after infection, the overlay and any non-adherent macrophages and promastigotes were removed. Miltefosine and OLPC solutions (35, 11.67, 3.89 and 1.30 µM) were applied to the infected macrophages and left for 72 h at 34°C and 5% CO_2_. Each experiment included 24 h and 72 h controls and an amphotericin B (Fungizone) control (included over a concentration range of 500, 167, 55.56 and 18.52 nM). The percentage of infected macrophages was counted upon microscopic evaluation (×100 magnification) compared with the untreated 72 h control. Sigmoidal curves were predicted using the four-parameter variable slope non-linear regression mode using Prism 9.0.2 (GraphPad software) and statistical differences were identified using a Student’s-*t*-test analysis.

### Antileishmanial efficacy against experimental CL

BALB/c mice were injected with 4 × 10^7^ stationary-phase *L. major* JISH118 or luciferase-expressing *L. major* Friedlin promastigotes subcutaneously in the shaven rump. Approximately 10 days later, a nodule of approximately 3 mm appeared, mice were allocated to their respective groups and drug treatment was started (for treatment information and group size, see Table 2). A dose of 35 mg/kg/day miltefosine and OLPC was selected to avoid fatal toxicity, which was previously observed at a dose of 40 mg/kg/day, particularly in the miltefosine-treated group.^14^ The mice were treated daily for 10 consecutive days. For the *L. major* JISH-infected mice, the size of the nodules was measured in two perpendicular directions using digital callipers recording the average diameter, whereas the parasite load in the mice infected with the luciferase-expressing *Leishmania* was followed and measured using an IVIS Lumina XRMS system (PerkinElmer, Hamburg, Germany). Briefly, in these mice 100 µL of d-luciferin was administered subcutaneously above the hind leg. Seven minutes after injection, the mice were anaesthetized with 2.5% isoflurane and another 3 min later they were placed in the imaging chamber where the bioluminescent signal was measured using exposure times varying between 1 s and 5 min, depending on signal intensity. The software package Living Imaging (PerkinElmer) was used to draw circular regions of interest (ROIs) to determine the signal intensity [or flux (ρ/s)]. Each experiment included an untreated control group (*n* = 5) and a positive-control group [paromomycin (intraperitoneally, IP), 50 mg/kg/day, *n* = 5] A blank control group (*n* = 3) composed of mice infected with the WT *L. major* JISH (that does not express luciferase) was equally included to reflect the background bioluminescence signal emitted by mouse skin. The mice were culled a day after the last drug administration and a circular disc of skin encompassing the nodule was collected and stored at −70°C until further processing. A one-way analysis of variances (ANOVA) with the Tukey *post hoc* test (*P* < 0.05) was performed to evaluate parasite load differences between treatment groups at the end of treatment using the GraphPad Prism software v9. The effect of treatment on the parasite load over time was evaluated with a repeated-measures ANOVA (Dunnett’s multi comparison test) (*P* < 0.05) was performed in SPSS v28.0.1.1 (IBM, Portsmouth, UK).

### Parasite load quantification in the skin

The parasite load in the skin was determined by quantitative PCR as described previously.^17^ Briefly, the infected skin was cut into smaller pieces on a glass microscope slide using a scalpel blade, after which they were transferred to a reinforced 2 mL tube with beads (Bertin Technologies) together with 1 mL of PBS. The tubes were placed in the Precellys Evolution (Bertin technologies) applying three cycles of 6500 rpm for 60 s separated by 1 min resting periods on ice. Fifty microlitres of the homogenate was submitted to DNA extraction using the Blood and Tissue extraction kit (QIAGEN) and eluted in 50 µL of sterile water. The DNA of the samples and the standard curve were subsequently used in a quantitative PCR (qPCR) using primers and a FAM-probe targeting the 18S *Leishmania* kinetoplast DNA (FP: 5′—CCAAAGTGTGGAGATCGAAG—3′, RP: 5′—GGCCGGTAAAGGCCGAATAG—3′ and probe: 6 FAM-ACCATTGTAGTCCACACTGC-NFQ-MGB). The standard curve was prepared by submitting 50 µL of the skin homogenate spiked with 1 × 10^8^*L. major* promastigotes to the same extraction procedure as the samples and 10-fold dilute.

The amplification reaction was performed in a 10 µL volume containing 1 µL of genomic DNA, each primer at 400 nM, 100 nM probe and 5 µL of the 2 ×  SensiFAST Probe mix (Bioline). PCR cycle conditions consisted of an initial denaturation step at 95°C for 5 min, 40 cycles of 95°C for 10 s and 60°C for 40 s. The samples were analysed in duplicate and a standard curve, a no-template control and a negative control were included in each run. The limit of quantification was established as 1000 *L. major* parasites per 50 µL of skin homogenate. A one-way ANOVA with the Tukey *post hoc* test (*P* < 0.05) was performed to investigate statistical differences in skin parasite load between the different treatment groups at the end of treatment.

## Results

### The antileishmanial activity of OLPC and miltefosine in the intramacrophage assay

To ensure broad-range antileishmanial activity, OLPC was evaluated against a range of intracellular amastigotes of both Old and New World *s*pecies and demonstrated EC_50_ values ranging from 3.63 to > 35 µM (Table 3). These values are similar to the EC_50_ values obtained for miltefosine, which ranged from 3.31 to >35 µM (Student’s *t*-test, *P* > 0.05), respectively.

**Table 3.** Activity of OLPC against intracellular *Leishmania* amastigotes

	*n*		*L. major* JISH118	*L. major* Friedlin	*L. tropica* HTD4	*L. aethiopica* KH	*L. mexicana* M379	*L. panamensis* Boynton	*L. amazonensis* HTD13	*L. braziliensis* M2903
Amphotericin B	1	EC_50_ (95% CI)	<0.03	0.04 (0.035–0.047)	< 0.03	< 0.03	< 0.03	< 0.03	0.17 (0.16–0.20)	< 0.03
(Fungizone^®^)		EC_90_ (95% CI)		0.07 (0.060–0.078)					0.48 (0.36–0.64)	
	2	EC_50_ (95% CI)	<0.03	< 0.02	< 0.03	0.088 (0.079–0.095)	0.038 (0.033–0.043)	0.012 (0.009–0.016)	0.11 (0.097–0.110)	0.002 (0.001–0.005)
		EC_90_ (95% CI)				0.14 (0.11–0.16)	0.11 (0.081–0.14)	0.033 (0.025–0.041)	0.22 (0.19–0.25)	0.17 (0.10–0.35)
Miltefosine	1	EC_50_ (95% CI)	20.97 (18.13–24.26)	30.81 (23.25–43.49)	11.15 (8.59–14.02)	11.26 (10.84—xx)	12.15 (xx—xx)	8.43 (7.02–10.02)	> 35	12.95 (10.97–15.51)
		EC_90_ (95% CI)	65.95 (48.76–98.65)	67.74 (xx—296.1)	35.49 (18.88–63.49)	18.43 (xx—22.46)	65.95 (48.76–98.65)	22.04 (16.38–32.43)		33.44 (19.60–52.02)
	2	EC_50_ (95% CI)	3.31 (2.90–3.76)	22.23 (19.22—xx)	16.23 (13.89–19.02)	11.66 (10.66–12.75)	12.98 (10.06–16.78)	9.09 (8.07–10.18)	> 35	14.78 (13.76–15.87)
		EC_90_ (95% CI)	15.64 (11.87–21.29)	35.65 (30.04–40.75)	xx	21.91 (xx—30.48)	60.20 (34.15–131.10)	24.84 (20.07–32.01)		29.29 (22.76–35.23)
OLPC	1	EC_50_ (95% CI)	6.99 (4.67–10.31)	11.43 (9.17–14.23)	1.78 (1.56–2.11)	3.42 (3.01–3.88)	27.81 (23.23—xx)	7.76 (6.66–9.03)	> 35	3.76 (2.87–5.30)
		EC_90_ (95% CI)	83.68 (38.18–286.00)	57.96 (36.77–103.70)	13.27 (9.44–19.63)	10.54 (9.02–14.35)	83.68 (38.18–286.00)	22.20 (16.89–30.79)		10.94 (xx—28.38)
	2	EC_50_ (95% CI)	1.22 (1.01–1.40)	3.63 (3.20–4.12)	11.7 (9.95–13.20)	4.03 (3.33–5.15)	25.74 (23.50–28.35)	7.02 (6.11–8.05)	> 35	5.46 (4.53–6.56)
		EC_90_ (95% CI)	8.08 (6.15–10.96)	15.64 (11.82–21.21)	41.19 (19.68–415.5)	13.20 (6.65–24.36)	50.61 (44.54–58.89)	27.25 (21.09–36.30)		24.39 (22.76–35.23)

EC_50_ and EC_90_ values in µM (95% CI). *n* = number of independent assays and each concentration was evaluated in quadruplicate in each independent assay. xx, Prism software was unable to generate this value based on the provided data.

More clinically relevant are the EC_90_ values that range from 11.9 to 68.7 µM (average of the EC_90_ values obtained in the two independent experiments) and 20.2 to 63.1 µM for OLPC and miltefosine, respectively. For all sigmoidal curves, the Hill slopes obtained for OLPC (other than those for *L. mexicana*) were lower compared with miltefosine, indicating an apparent trend it might be less potent with small increases above the EC_50_ concentration although without significant differences.

### The efficacy and safety of OLPC and miltefosine in an experimental CL model

**Figure 1.**
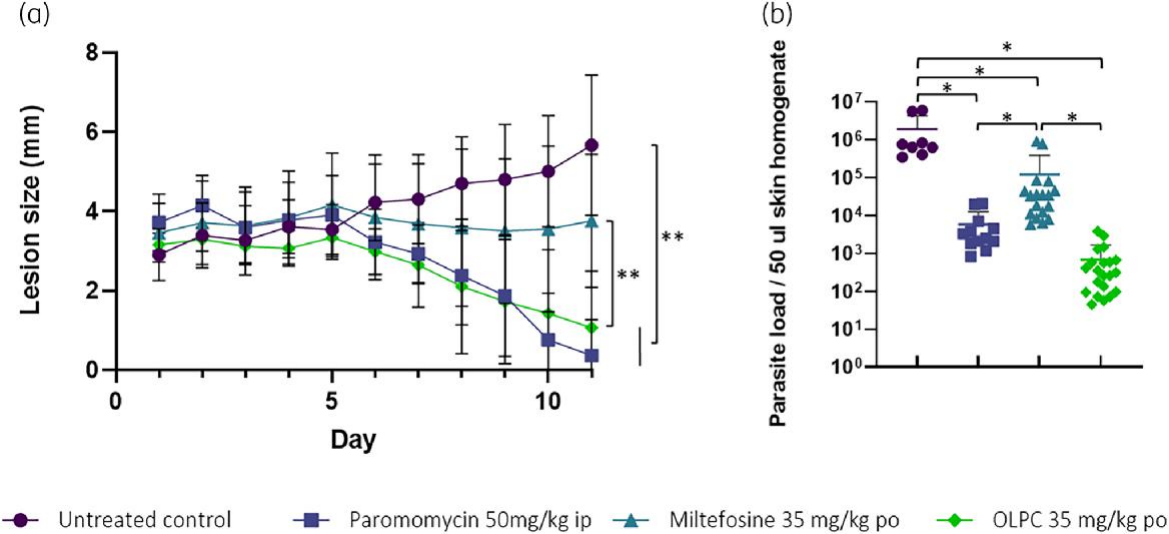
Antileishmanial efficacy of OLPC and miltefosine (oral administration, 35 mg/kg once daily for 10 days) in an Old World CL model (*L. major* JISH118 infection of BALB/c mice). (a) The average lesion size over time (*n* = 5). (b) The parasite load in the skin on day 11 was confirmed using qPCR. *P < 0.05, one-way ANOVA; **P < 0.05, repeated-measure ANOVA. This figure appears in colour in the online version of *JAC* and in black and white in the print version of *JAC*.

In the CL model, a daily administration of 40 mg/kg OLPC and miltefosine was reported to induce signs of toxicity.^14^ To avoid these, our first experimental study design aimed at evaluating both the antileishmanial efficacy and safety of a 10 day oral dosing regimen of 35 mg/kg/day. Throughout the treatment duration with the 35 mg/kg dose, the mice in the OLPC and miltefosine group did not display signs of suffering recognized as piloerection, hunched posture, ruffled fur or weight loss greater than 10% compared with weights prior to the study, indicating this dosing regimen was well tolerated. Moreover, OLPC administered orally at a dose of 35 mg/kg once daily for 10 days was able to significantly reduce the lesion size to a similar extent as the positive control (paromomycin sulphate, IP, 50 mg/kg/day, repeated-measures ANOVA, *post hoc* Tukey, *P* < 0.05) (Figure 1a). In contrast, the administration of miltefosine (same dose and regimen as OLPC) only resulted in a halt of the lesion size progression and was unable to decrease the lesion diameter (no statistically significant difference with untreated control group, repeated-measures ANOVA, *post hoc* Tukey, *P* > 0.05). The parasite burden in the skin reflects this trend, with a statistically significantly lower parasite load recovered from the skin for the OLPC and miltefosine treatment compared with the untreated control group, with the former inducing the most pronounced reduction (Figure 1b).

A follow-up study was designed with three aims: (i) to ascertain the relationship between the drug dose and the observed antileishmanial activity dose–response assessment; (ii) to establish a rate-of-kill for the effective doses; and (iii) to compare the antileishmanial performance of OLPC formulations with rapid and slow-release profiles developed to facilitate the pharmaceutical development process.

**Figure 2.**
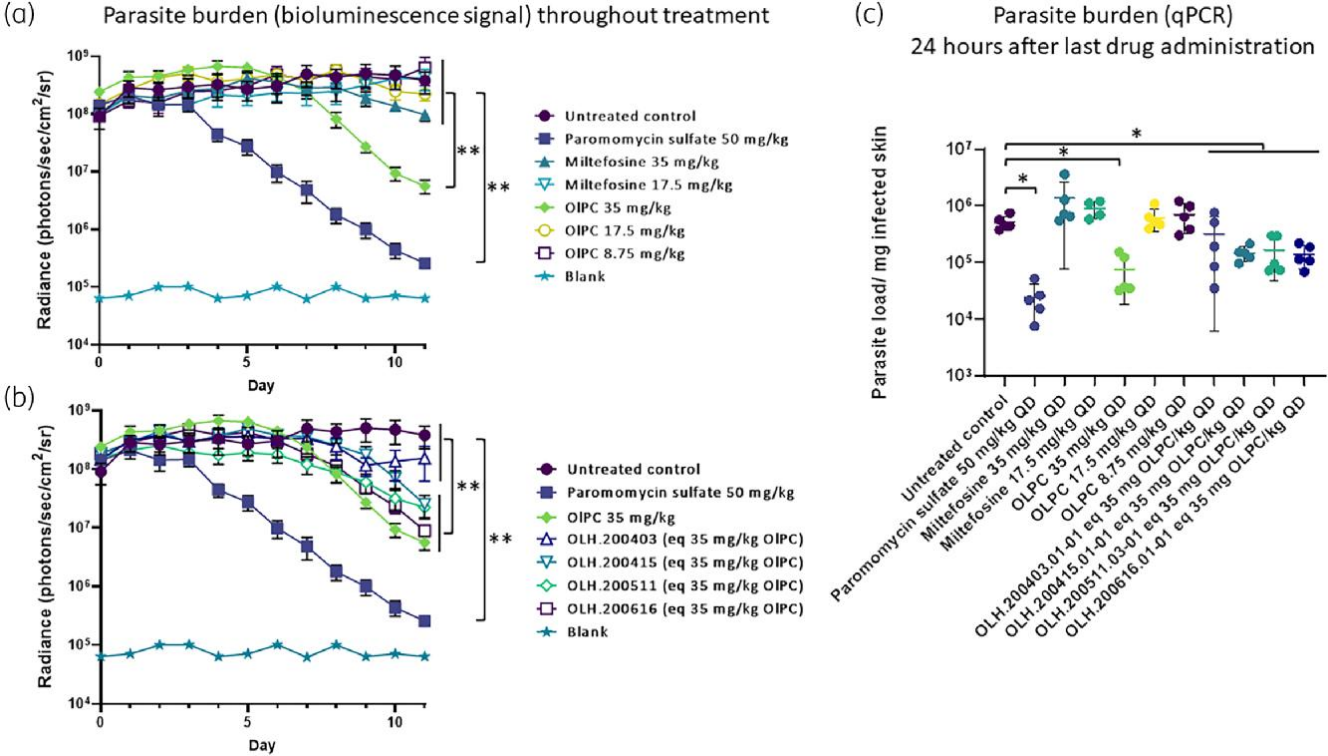
The antileishmanial efficacy of oral miltefosine and OLPC (once daily for 10 days) in an Old World CL model (Ppy RE9H + *L. major* Friedlin infection of BALB/c mice). (a) Dose–response titration of the antileishmanial efficacy. The parasite load, as indicated by *in vivo* imaging of bioluminescent parasites in the infected rump skin over time. (b) OLPC formulations versus OLPC as such. The parasite load, as indicated by *in vivo* imaging of bioluminescent parasites in the infected rump skin over time. (c) The parasite load in the skin on day 11 was confirmed using qPCR. QD, once daily; *P < 0.05, one-way ANOVA; **P < 0.05, repeated-measure ANOVA. This figure appears in colour in the online version of *JAC* and in black and white in the print version of *JAC*.

Using the bioluminescence signal emitted by the luciferin-expressing *L. major* Friedlin strain, viable parasite loads in the skin were measured with a limit of detection of approximately 20 000 parasites.^18^ The study demonstrates a superior activity of the two fast- [OLPC with lactose (OLH.200511) or cellulose carrier (OLH.200616)] and one slow diffusion-controlled silica carrier [(OLH.200415) test formulation as measured by a significantly greater bioluminescence signal and thus parasite load decrease when compared with the untreated controls (Figure 2; OLH.200511, OLH.200616 and OLH.200415 compared with untreated control group, repeated-measures ANOVA, *post hoc* Tukey, *P* < 0.05). There was also a clear lack of antileishmanial activity for the OLPC concentrations below 35 mg/kg/day (i.e. 17.5 and 8.75 mg OLPC/kg/day) and the miltefosine (35 and 17.5 mg/kg/day)-treated groups (no statistically significant difference with untreated control group, repeated-measures ANOVA, *post hoc* Tukey, *P* > 0.05).

To identify whether the pharmaceutical formulations impact the antileishmanial efficacy, the average percentage of reduction in skin parasite load upon each administered dose [from Day 7 (sixth dose) to Day 11 (tenth dose)] resulted in the following rank order: OLPC 35 mg/kg (57%) > paromomycin (51%) > OLH.200616 (50%) > OLH.200415 (38%) > OLH.200511 (34%) > OLH.200403 (10%). Even so, OLH.200415 demonstrated a steeper slope from dose 9 to 10 (62%). This indicates that OLPC administered as such more effectively removed the *Leishmania* parasites from the skin, followed by the cellulose-containing formulation (OLH.200616), the extruded mesoporous silica particles (OLH.200415), the lactose combination (OLH.200511) and finally the silica particles that were loaded using ethanol (OLH.200403).

## Discussion

Miltefosine was first brought to the market as an anticancer drug (Miltex^®^) in the 1990s. In parallel, research was ongoing to evaluate its potential as an antileishmanial drug and led to its approval to treat VL in the early 2000s and later also for CL.^19^ Today, the availability of miltefosine, one of the key drugs in an already fragile treatment arsenal for CL, is problematic, highlighting the urgent need for additional effective antileishmanial drugs.^8,9,20^ As a close analogue of miltefosine with potent activity against VL,^11,12,14^ OLPC is a promising candidate for development as CL treatment.^14^ Nevertheless, the different sites of primary infection of the parasites in VL and CL make different PK demands on the compounds; in addition, the subtle structural differences between miltefosine and OLPC could potentially result in differences in pharmacokinetic and pharmacodynamic parameters and thus antileishmanial efficacy.

In our study, OLPC demonstrated a potent activity across a range of CL-causing species consistent with values reported previously.^11,21^ Nevertheless, subtle differences in EC_50_ values can be observed notably for *L. mexicana* and *L. tropica*, which could be attributed to variability in assay conditions (both inter- and intra-laboratory) and biochemical and molecular differences across subspecies.^11,22,23^ Overall, the *in vitro* antileishmanial activities of OLPC and miltefosine are similar and the sigmoidal curve of both drugs indicated a steep Hill slope, implying that a small increase in drug concentration can produce a large increase in antileishmanial activity. Moreover, when using 40 µM miltefosine as the highest test concentration, toxicity to the macrophages was noticeable, indicating a low selectivity index and a narrow therapeutic window. This was equally observed for the same concentration of OLPC (data not shown).

In the experimental CL models, OLPC displayed a statistically superior antileishmanial profile to miltefosine. In our first study utilizing mice infected with *L. major* JISH parasites, OLPC at 35 mg/kg/day significantly reduced the lesion size and skin parasite burden to the same extent as the positive-control paromomycin 50 mg/kg/day (IP). However, at an equivalent dose, miltefosine suppressed growth of the lesion but was unable to reduce the lesion size. Introducing a luciferase-expressing *L. major* strain, the impact of each drug dose on the parasite burdens in the skin can be assessed to dissect the dose–response of OLPC and miltefosine in the *in vivo* model.^24^ It was apparent that miltefosine was unable to significantly impact the parasite burden (measured by qPCR and bioluminescence signal) in the dermal layers of the skin at a dose of 35 mg/kg/day in contrast to the observations of the first study where a significant difference in lesion size and parasite load (measured by qPCR) compared with the untreated control was observed. One possible explanation is that the Ppy RE9H + *L. major* Friedlin strain is less susceptible to miltefosine as indicated by an average *in vitro* EC_50, JISH118_ of 12 µM compared with EC_50, Ppy RE9H + Friedlin_ of 27 µM. Nevertheless, OLPC demonstrated a significantly more rapid reduction of *Leishmania* parasites in the skin compared with miltefosine. This trend needs to be confirmed in clinical studies but could potentially reduce the lengthy treatment duration currently required for miltefosine.

Differing susceptibility of the *L. major* strains used in the experimental models to OLPC and miltefosine could also explain why OLPC is more efficient at killing parasites in the dermal layer of the skin in the mouse model than miltefosine. It is further worth highlighting that in contrast to paromomycin, where the first decrease in parasite load is evident after the second dose (corresponding to the bioluminescence signal Day 3), the antileishmanial activity of OLPC and miltefosine in the mouse model is only apparent after 5 and 8 doses (bioluminescence signal Days 6 and 9), respectively. This indicates that when administered orally both alkylphosphocholines require time to reach sufficiently high concentrations in the phagolysosome of the *Leishmania*-infected cell located in the dermal layers of the skin. This is expected given the long half-lives of OLPC and miltefosine (50 h^12^ and 84 h^25^ in rodents, respectively), indicating steady-state concentrations are only expected to be reached after 10 and 18 days (or 5 × *t*_½_ assuming a one-compartment system) of drug administration. Even so, previous reports highlight efficient distribution of miltefosine to tissues, notably the skin.^26^

Whilst studies measuring OLPC concentrations in the various organs are lacking, a study conducted in hamsters reported a distribution volume of 3.026 L/kg, suggesting good tissue distribution. Expanding this to patients, the concentration of miltefosine in a skin biopsy removed after 21 days of post kala-azar dermal leishmaniasis (PKDL) treatment indicated a concentration range from 21 to 52 µg of miltefosine per gram of skin tissue.^27^ Given that this is above general concentrations found in the plasma, miltefosine indeed seems to concentrate in the skin. Moreover, these concentrations are in the same order as the EC_90_ values obtained for miltefosine (52 µM, ∼21.3 µg/mL), suggesting they are adequate for parasite killing. The *in vitro* model, which contains only infected macrophages in medium, is a simplistic representation of a *Leishmania* infection and is not representative of the complex skin-macrophage-*Leishmania* microenvironment encountered in patients. It is thus important to remember that *Leishmania* parasites are situated in the phagolysosome of the macrophage and little is known about the local distribution of the drug in the microenvironment surrounding the parasite, especially for drug molecules that are structurally similar to cell membrane components. In an attempt to elucidate drug concentrations in the different compartments, the amount of miltefosine inside peripheral blood mononuclear cells was established and indicated an intracellular concentration exceeding the concentration found in the plasma.^28^ These findings merit further investigations more particularly in the dermal layers of the skin, which is the relevant microenvironment.

Development of an oral OLPC formulation presents challenges inherent to its alkylphosphocholine structure. For example, the low melting point increases particle stickiness, the double bond is sensitive to oxidation and the hygroscopic character potentially affects the stability of the final formulation. Lactose and microcrystalline cellulose are cheap and commonly used excipients in capsules whereas the encapsulation of OLPC in the mesoporous silica was chosen to provide enhanced stability to the final formulation and modify the release profile to circumvent adverse effects similarly to those observed in patients receiving miltefosine treatment whilst maintaining the antileishmanial activity. Of the different formulations, OLPC administered as such demonstrated the highest rate of kill alongside the rapid-release cellulose and lactose formulation and the slow-release extrusion loaded silica particles. The mice treated with the solvent-loaded OLPC-silica particles demonstrated a slower reduction of the bioluminescent signal. Even so, the parasite load measured by qPCR was similar to the other test formulations and significantly lower than the untreated group (one-way ANOVA, *P* < 0.05) but demonstrated a notable higher standard deviation than the other test formulations. Given the additional cost of the silica particles and the lack of a superior antileishmanial efficacy, simple excipients were selected for further studies.

Alternatively, the development of a topical formulation would reduce or avoid systemic drug exposure whilst maximizing local skin drug exposure. Previous work investigating the potential of topical miltefosine formulations to treat CL demonstrated that only negligible concentrations of drug were present in the skin when applied in a range of vehicles.^29^ None of those formulations resulted in a lesion size reduction when applied to CL nodules in the BALB/c mouse model. Given the similar physicochemical properties between OLPC and miltefosine, a topical OLPC formulation with commonly used low-cost excipients is unlikely to result in sufficiently high skin drug concentrations that are required to efficiently clear cutaneous *Leishmania* parasites.

Together, our results show that OLPC is a promising drug for the oral treatment of CL but further research is required to (i) elucidate local skin distributions as they might be relevant to clinical outcomes; and (ii) evaluate the antileishmanial efficacy against other *Leishmania* species, notably New World species.
